# In vitro SELEX and application of an African swine fever virus (ASFV) p30 protein specific aptamer

**DOI:** 10.1038/s41598-024-53619-7

**Published:** 2024-02-19

**Authors:** Changchun Hu, Shuo Li, Jie Zhou, Dan Wei, Xueying Liu, Zhu Chen, Hongquan Peng, Xun Liu, Yan Deng

**Affiliations:** 1 Hunan Key Laboratory of Biomedical Nanomaterials and Devices, Hunan University of Technology, Hunan, 412007 Zhuzhou China; 2 Institute for Future Sciences, University of South China, Changsha, Hunan China; 3 Hengyang Medical School, University of South China, Hengyang, 421001 Hunan China; 4 Department of Nephrology, Kiang Wu Hospital, Macau, SAR China; 5 Department of Nephrology, The Third Affiliated Hospital of Sun Yat-Sen University, Guangzhou, China

**Keywords:** DNA, Enzymes, Analytical biochemistry, Sensors and probes, Isolation, separation and purification, Infectious-disease diagnostics

## Abstract

The African swine fever virus (ASFV) has caused severe economic losses in the pig industry. To monitor ASFV spread, the p30 protein has been identified as an ideal infection marker due to its early and long-term expression during the ASFV infection period. Timely monitoring of ASFV p30 enables the detection of ASFV infection and assessment of disease progression. Aptamers are an outstanding substitute for antibodies to develop an efficient tool for ASFV p30 protein detection. In this study, a series of aptamer candidates were screened by in vitro magnetic bead-based systematic evolution of ligands by exponential enrichment (MB-SELEX). An aptamer (Atc-20) finally showed high specificity and affinity (K_d_ = 140 ± 10 pM) against ASFV p30 protein after truncation and affinity assessment. Furthermore, an aptamer/antibody heterogeneous sandwich detection assay was designed based on Atc20, achieving a linear detection of ASFV p30 ranging from 8 to 125 ng/ml and a detection limit (LOD) of 0.61 ng/ml. This assay showed good analytical performances and effectively detected p30 protein in diluted serum samples, presenting promising potential for the development of ASFV biosensors.

## Introduction

African swine fever virus (ASFV) is the sole member of the Asfarviridae family; it is an enveloped and double-stranded DNA virus that causes a high-mortality disease named African swine fever (ASF) in swine^1^. Highly virulent isolates of ASFV have been found to result in 100% mortality in both domestic and wild swine within 4–15 days^2^. The infected animals display symptoms such as high fever, respiratory changes, inappetence, and inactivity, and eventually die from shock^3^. ASFV originates from Africa, first spread in 1957, and has subsequently been found repeatedly in Eurasia and American countries^4–6^. ASFV has caused severe economic losses in the pig industry due to the lack of effective vaccines or available treatment protocols^7^.

Current ASFV detection methods mainly include gene detection-based methods and the detection of antibodies^8–10^. Gene detection based on quantitative polymerase chain reaction (qPCR) has become the gold standard for ASFV detection due to its high sensitivity. However, this method requires a time-consuming sample pre-treatment process and can only be performed by professional operators. Despite the high sensitivity, some false positive and false negative results influence the accuracy^11,12^. Nonetheless, antibody detection provides a simpler and easier approach. The analytical performance of these antibody-based methods highly depends on the quality of antibodies or recombinant antigens, and their production is relatively complicated. Aptamers are considered an ideal candidate for developing diagnostic tools for ASFV due to their low cost, high affinity, and unique chemical features^13–15^.

Aptamers are single‑stranded DNA or RNA that specifically recognize a particular target, and are screened through systematic evolution of ligands by exponential enrichment (SELEX) from an in vitro random nucleotide library^16,17^. SELEX generally includes three critical processes, namely binding between random oligonucleotide library and the target, separation between bound and un-bound nucleotides, and amplification of bound nucleotides. In addition, single DNA or RNA can form unique three-dimensional shapes under special conditions, which promote hydrogen bonding and hydrophobic interactions, as well as electrostatic interactions with diverse target molecules, resulting in a high affinity^18^. In addition to the attractive chemical properties of aptamers, they are also easily synthesized and chemically modified, with higher chemical and thermal stability than antibodies^19^. Moreover, virus biosensors based on aptamers also exhibit promising application potential due to their high sensitivity, low cost, and good stability^20–23^.

The ASFV diagnostic target proteins are conserved and immunogenic, including p54, p72, p30, etc.^24–26^. As a structural protein, the p30 protein, which is encoded by ORF CP204L, is located in the inner membrane of the viral envelope. Its expression is generally observed from about 2 to 4 h post-infection and is persistent during the infection cycle in cells^27–29^. Besides, research revealed that the viral protein p30 could serve as an indicator of acute ASFV infection in animals^26^. Therefore, the p30 protein may be applied for the early detection of ASFV, providing a tool to control the spread of ASFV. The p30-specific aptamer can be utilized to develop an early, low-cost, and point-of-care ASFV detection assay. In this work, aptamers targeting ASFV p30 protein were selected using a magnetic-based selection strategy (MB-SELEX). After 7 rounds of SELEX and further aptamer truncation and identification, an optimal Atc-20 truncated aptamer was obtained. Furthermore, an antibody/aptamer sandwich colorimetric assay was fabricated for ASFV p30 protein detection, demonstrating outstanding application potential for Atc-20 as an ASFV p30-specific probe.

## Results

### In vitro SELEX of ASFV specific aptamer

**Figure 1:**
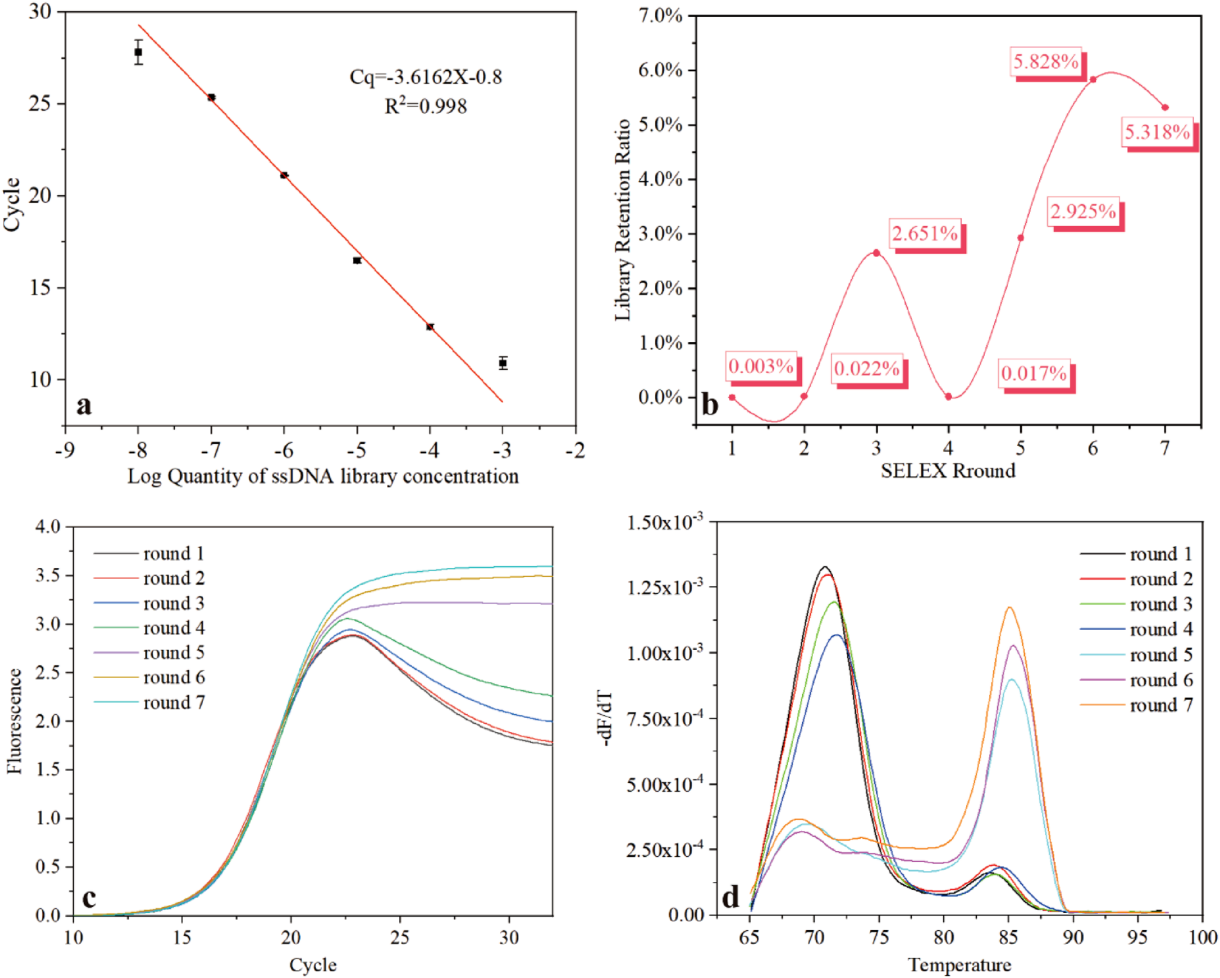
(**a**) The fitted standard quantitative curve, the log quantity of library concentrations (10^–3^–10^–8^ μM) was linearly correlated with Cq value, R^2^ = 0.998; the error bars correspond to the standard deviation from triplicate measurements (n = 3). (**b**) The retention ratio of rounds 1–7. (**c**) The amplification curves for purified products of rounds 1–7. The curves were translated on the x-axis to improve the contrast. (**d**) The melting curves for purified products of rounds 1–7. The curves displayed fluorescent variation following increased temperature. (qPCR melting program: 95 °C for 10 s, 65 °C for 60 s, 97 °C for 1 s). The qPCR experiment was conducted on a Light Cycle^®^96 instrument (Roche, Switzerland).

The ASFV p30 protein was subjected to 7 rounds of MB-SELEX selection. The linear dependence relationship of the log quantity of ssDNA concentration and Cq value is shown in Fig. 1a. As shown in Fig. 1b, the retention ratio showed an increased trend in general. The unusual decline in the third round might be attributed to the addition of swine serum, which contained a complicated mix of ingredients. Indirectly, it also indicated the successful removal of nonspecific aptamers. The retention ratio reached higher values during the 6th and 7th rounds.

The qPCR amplification and melting curves for purified products from each round were analyzed to comprehensively assess the SELEX progression Fig. 1c,d. The amplification curve, displayed in Fig. 1c, revealed an apparent fluorescence drop in rounds 1–4. This drop presented a distinct decrease after the 5th round and the amplification curve plateau emerged, corresponding to the random and non-specific oligonucleotide reduction with persistent SELEX. Similarly, as shown in Fig. 1d, the decreased ds-DNA hetero-duplex (Tm at 70–72 °C) and increased homo-duplex (Tm at 82–87 °C) resulted in a gradual drop and increase in the melting peak around 71 °C and 85 °C, respectively. These phenomena confirmed that the ssDNA library was already enriched in rounds 5–7 following the convergence of ssDNA species^30^. Considering that the retention ratios of rounds 6–7 showed no further increase, the SELEX was terminated in the 7th round.

### Screening aptamer candidates of high binding affinity

**Figure 2:**
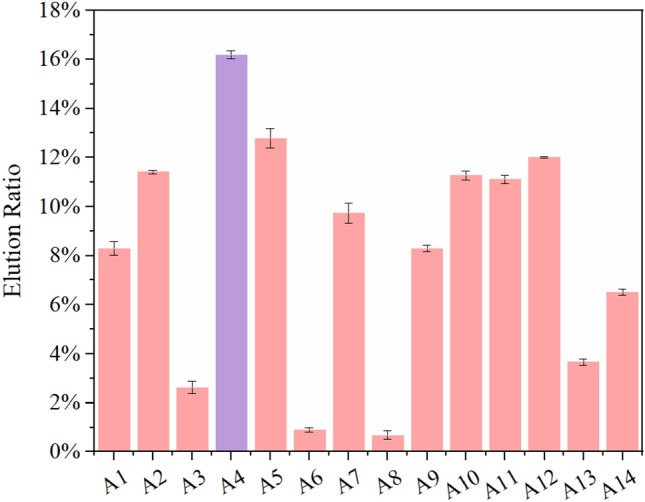
The elution ratios for pt A1–14; Apt A4 (lavender color) presented the highest elution ratio as well as the highest affinity to p30 protein; the error bars correspond to the standard deviation from triplicate measurements (n = 3).

The high-throughput sequencing showed that the top 14 sequences accounted for more than 60% of the results, as shown in Table S1 (the sequencing datasets are available in the Sequence Read Archive (*SRA*) repository, [SRA data: PRJNA990511]). Their second structures were predicted, revealing a consistent loop structure on both ends and a varying stem-loop structure at 20–60 nt (Figure S1). Subsequently, the sequences of Apt A1–14 were synthesized to verify their binding affinity to the p30 protein. After incubation with 40 μl p30 magnetic beads (MBs) for 30 min, the elution amounts of Apt A1–14 were determined, as summarized in Fig. 2. Apt A4 presented the highest elution amount and the highest affinity to p30 protein.

### Aptamer truncation and K_d_ measurement

**Figure 3:**
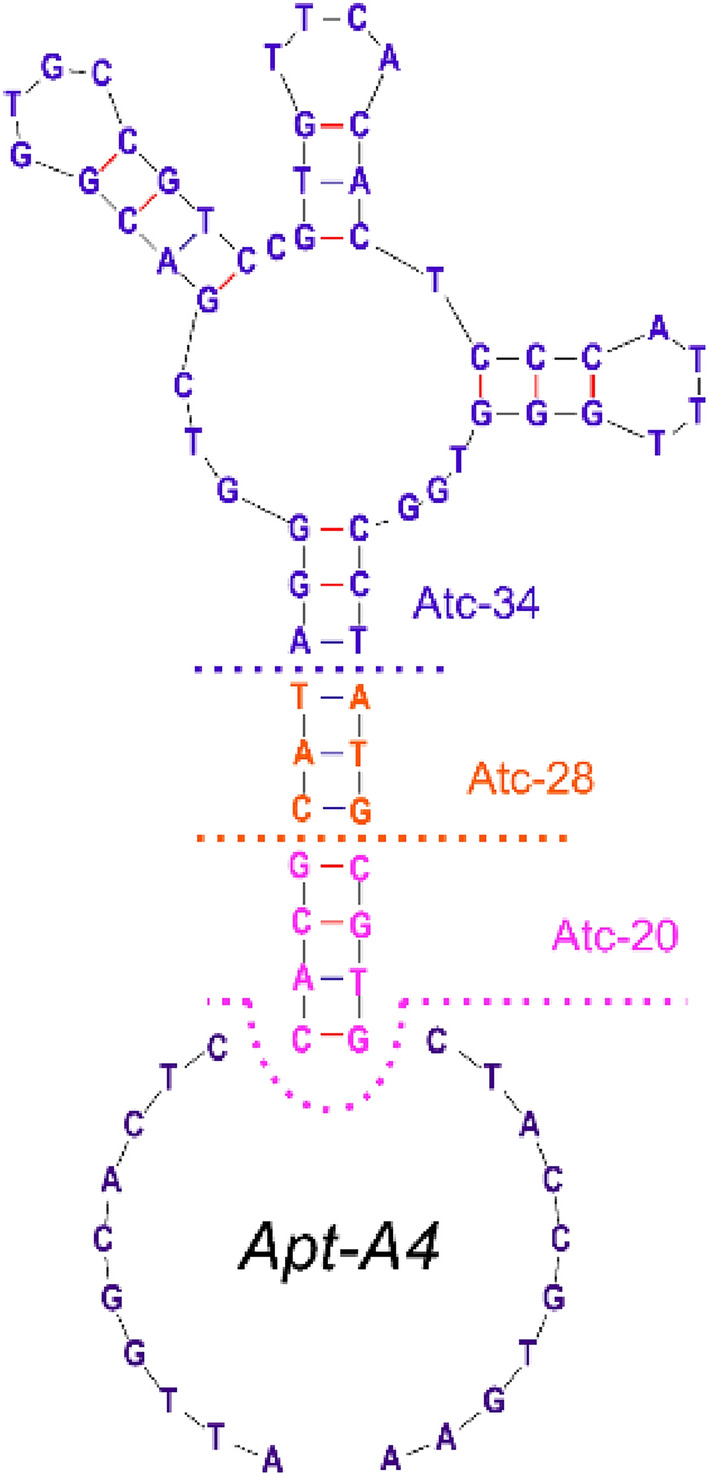
Apt A4 was symmetrically truncated from the 3′ and 5′ ends at 10, 14, and 17 nucleotides. The affinity to the p30 protein started to decrease when 28 nt nucleotides were cut off.

**Figure 4:**
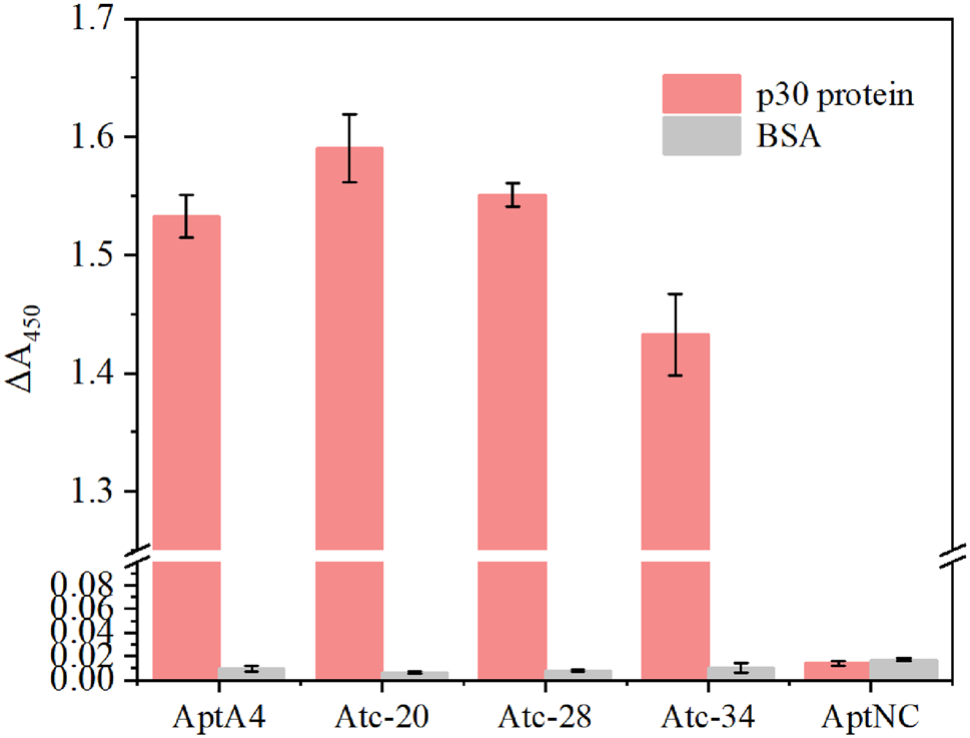
The enzyme-linked aptamer adsorbed assay showed the binding affinity for the truncated aptamers. Absorbances were measured at 450 nM, Atc-20, and Atc-28, exhibiting higher absorbance values, which confirmed their binding affinity to the p30 protein. Moreover, all the control groups exhibited low absorbance values and showed no binding affinity. The absorbance in this research was measured by Synergy HTX Multimode Reader. The error bars correspond to the standard deviation from triplicate measurements (n = 3).

The binding affinity can be determined by qPCR quantification of aptamers, providing a simple and low-cost method. However, the truncation of aptamers resulted in the deletion of the primer region, which led to the repeat design of primers and determination of qPCR standard curves and compromised reliability. Thus, the enzyme-linked aptamer adsorbed assay was employed to verify the binding affinity of truncated aptamers. The second structure of Apt A4 was analyzed and 20, 28, and 34 nucleotides were truncated (Fig. 3), which were named Atc-20, 28, and 34, respectively. The truncated aptamers were modified with biotin at the 5′ end to conduct the enzyme-linked aptamer adsorbed assay, as shown in Fig. 4. The truncated aptamers and AptA4, which were incubated and bound to the p30 protein, both exhibited strong absorbance signals. In contrast, weak signals were obtained after incubation with the BSA protein. Protein binding experiments with p30 protein and BSA revealed that AptA4 and the truncated aptamer had an affinity for p30 protein, but not for BSA protein.

**Figure 5:**
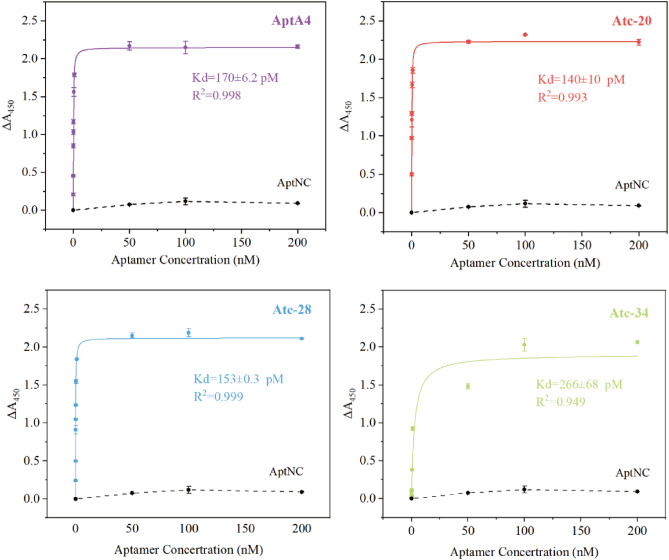
The affinity curve fitting for AptA4, Atc-20, 28, and 34. The p30 proteins were detected at 5 μg/ml and concentrations of aptamers were 20 pM–200 nM. The dashed line indicates the control group of AptNC. The error bars correspond to the standard deviation from triplicate measurements (n = 3).

Furthermore, non-linear fitting was performed on the above enzyme-linked aptamer experimental data to determine the binding curves, as shown in Fig. 5. The binding curves of aptamer AptA4 and the other truncated aptamers showed a clear concentration dependence, which was not observed in the binding curves of the control aptamer AptNC. The Kd values for Apt A4, Atc-20, 28, and 34 were determined as 170 ± 6.2 pM, 140 ± 10 pM, 153 ± 0.3 pM, and 266 ± 68 pM respectively. The binding affinity of the aptamer for the p30 protein changed as the number of truncated nucleotides increased. Atc-20 and Atc-28 demonstrated a slight increase in binding affinity when compared to Apt A4, indicating successful truncation. However, the affinity of Atc-34 was lower than the original aptamer, which suggests that a stem-like structure of sufficient length is required to maintain aptamer affinity and that over-truncation compromises the binding affinity.

### Analytical performance of antibody/aptamer sandwich assay

**Figure 6:**
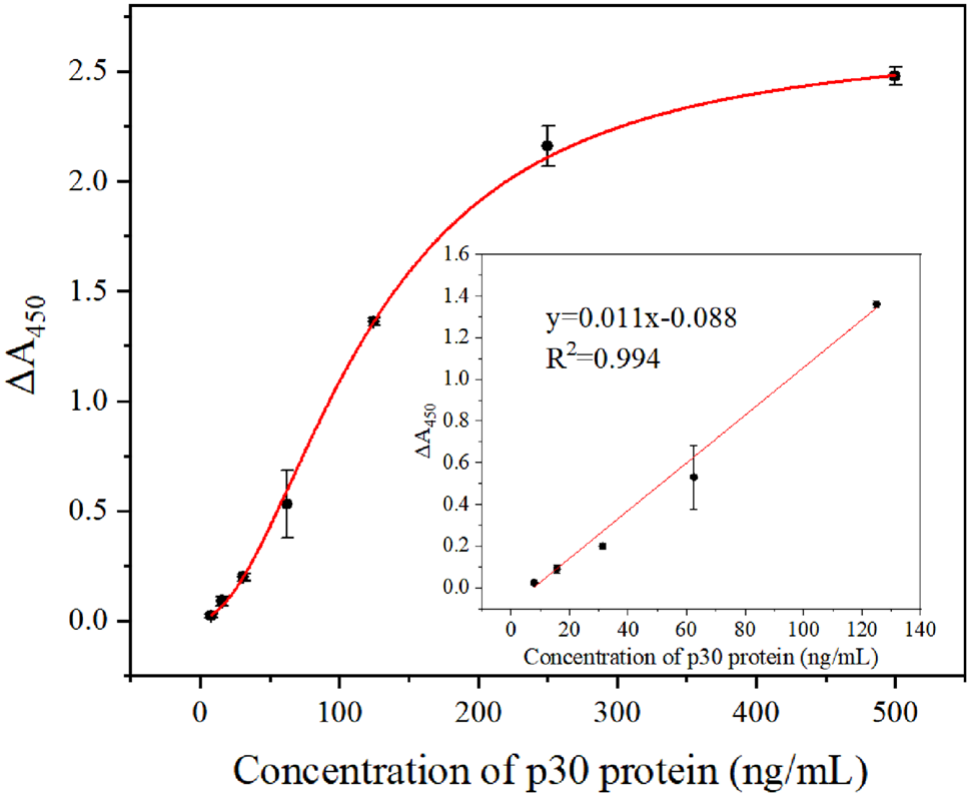
Analysis of ASFV p30 protein at the concentrations of 7.8, 15.6, 62.5, 125, 250, and 500 ng/ml. Inset: the calibration curve for absorbance versus concentrations of p30 protein. (y = 0.011x − 0.088, R^2^ = 0.994). The error bars correspond to the standard deviation from triplicate measurements (n = 3).

The enzyme-linked aptamer adsorbed assay exhibited a certain analysis capacity for ASFV p30 protein. Furthermore, an antibody/aptamer sandwich assay was developed using p30 monoclonal antibodies and Atc-20, in which the p30 monoclonal antibodies acted as the capture element and ATC-20 as the recognition element. ASFV p30 proteins were analyzed over a concentration range of 8–500 ng/ml, as displayed in Fig. 6. The absorbance of p30 protein increased progressively as the concentration was increased, showing a linear dependence within the concentrations 8–125 ng/ml. The LOD was calculated as 0.61 ng/ml following the formula 3.3 times blank standard deviation (n = 5) divided by the slope of the linear fitting equation.

### Specificity and practicability analysis of the antibody/aptamer sandwich assay

**Figure 7:**
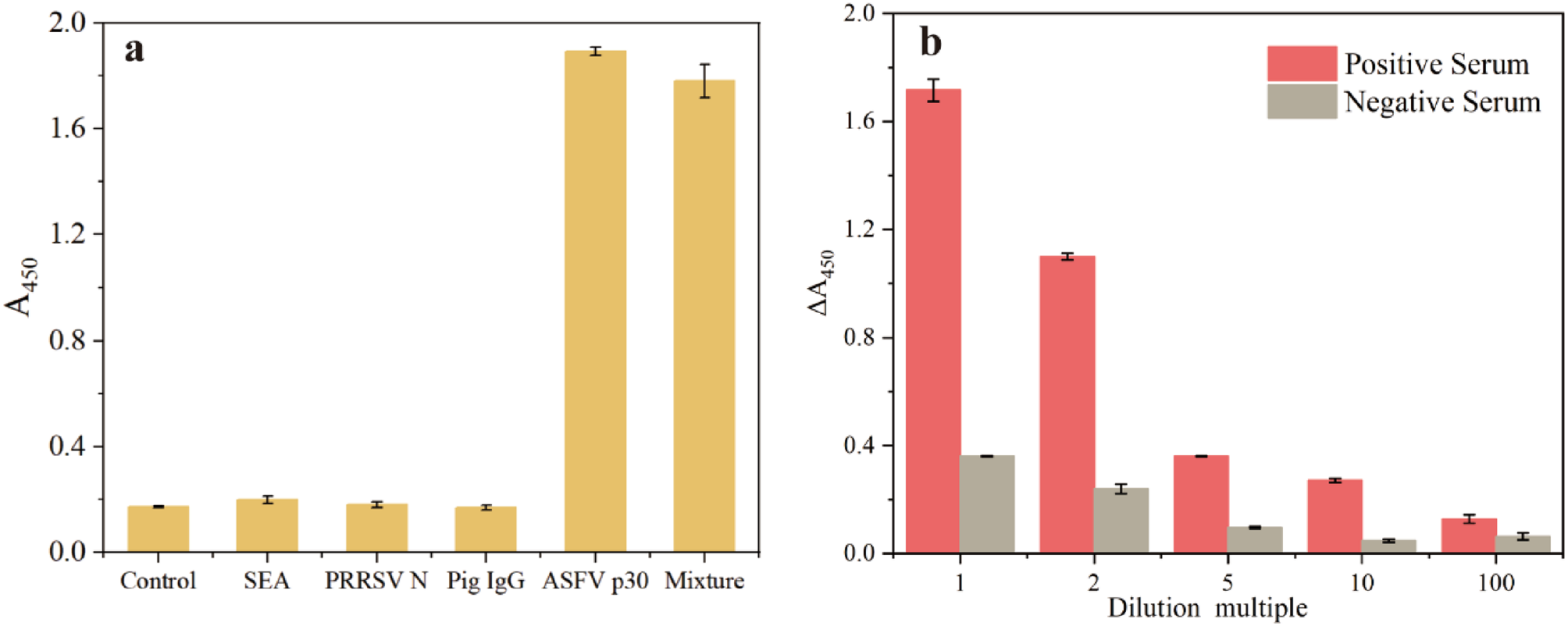
(**a**) Specificity of sandwich assays. ASFV p30 protein was detected at a concentration of 0.2 μg/ml and the other protein at 2 μg/ml; the mixture group consisted of 0.2 μg/ml and 2 μg/ml of other proteins. (**b**) The ASFV serum sample analysis of the sandwich assays. The serums were diluted with 0.01 M PBS. The error bars correspond to the standard deviation from triplicate measurements (n = 3).

The specificity of this assay was evaluated with tenfold concentrations of proteins that may appear concurrently with the p30 protein to determine its discriminative ability for complex samples. As seen in Fig. 7a, the non-specific signals for porcine reproductive and respiratory syndrome virus recombinant N protein (PRRSV N), and staphylococcus aureus enterotoxin type A recombinant protein (SEA), pig immunoglobulin G (IgG) samples were very low and close to the control group. In addition, the ASFV p30 and mixture groups exhibited strong signals. A slight decrease in signal was observed in the mixture group due to the presence of interfering proteins that may hinder the capture of p30. The ASFV serum sample analysis is shown in Fig. 7b. The positive serum sample exhibited a several-fold higher signal compared to the negative sample in different dilution ratios, which indicated that the sandwich assay effectively distinguished ASFV-containing samples from negative serum under suitable dilution. However, the results for the negative serum presented some noise signals due to complex constituents. Considering the potential influence of these findings on ASFV detection, appropriate signal processing (determining the difference or ratio) could be beneficial for the practical application of this assay. Moreover, the diluted serum samples were evaluated by the standard addition method. Three tenfold diluted serum samples were spiked with ASFV p30 standard solution to achieve concentrations of 10, 20, and 40 ng/ml, respectively. As shown in Table 1, the recovery ratios were calculated as 103%, 128%, and 106% for concentrations 10 ng/ml, 20 ng/ml, and 40 ng/ml, respectively; a satisfactory RSD ranging from 1.55 to 2.36% (n = 3) was obtained. These results confirmed the excellent specificity and potential practicability of the sandwich assay.

**Table 1:** Recovery ratio of developed antibody/aptamer assay in 10% serum samples.

Spiked concentration (ng/ml)	Detection concentration (ng/ml)	Recovery ratio (%)	RSD (%)
10	10.33	103	1.55
20	25.68	128	2.36
40	42.62	106	2.33

## Discussion

MB-SELEX constitutes a simple and cost-efficient method of screening. Moreover, the combination of MB-SELEX and qPCR provided a higher aptamer screening efficiency. Herein, the ASFV p30 protein-coated MB was used and the specific aptamer against ASFV p30 protein was successfully screened after 7 rounds of SELEX. The k_d_ value for the selected aptamer was measured as 170 ± 6.2 pM. Moreover, the aptamer was truncated to increase the binding affinity to the p30 protein, and a lower K_d_ value of 140 ± 10 pM was obtained with 20 nt nucleotide deletion. The antibody/aptamer sandwich assay was found to possess high specificity and practicability. As a capture element, the aptamer showed good performance. However, when the aptamer was immobilized on a plate as a capture element, the protein capture efficiency significantly decreased, which hindered the detection of ASFV. Follow-up studies should explore more feasible aptamer immobilization solutions or modification of aptamers according to their binding to proteins for wider application. Current methods of ASFV detection focus on gene and ASFV antibody detection, and antigen detection assays are infrequent. The screened ASFV p30 protein-specific aptamer can potentially be applied in the development of ASFV biosensors and in early ASFV detection.

## Methods

### Material

All oligonucleotides (HPLC purified) were synthesized and modified by Sangon Biotechnology Co., Ltd. (Shanghai, China). D-PBS buffer, 20× PBS buffer, 5× TBE buffer, RNase-free ddH_2_O, Tween-20, Bovine Serum Albumin (BSA), HRP-labeled Streptavidin (SA-HRP), and EL-TMB Chromogenic Reagent kit were purchased from Sangon Biotechnology Co., Ltd. (Shanghai, China). ASFV p30 recombinant protein, p30 monoclonal antibody, porcine reproductive and respiratory syndrome virus (PRRSV) recombinant N protein, staphylococcus aureus enterotoxin type A recombinant protein (SEA), and pig immunoglobulin G (IgG) were purchased from Aiyi Biotechnology. (Nanjing, China). 6× His tag peptides were bought from Synpeptide Biotechnology Co., Ltd. (Nanjing, China). Swine serum was sourced from Solarbio Science & Technology Co., Ltd. (Beijing, China). NHS (*N*-hydroxysuccinimide)-Activated Magnetic Beads (NHS-MB) were purchased from Beaver Biosciences Inc. (Suzhou, China). Taq PCR Master Mix and ChamQ Universal SYBR qPCR Master Mix were purchased from Vazyme Biotechnology Co., Ltd. (Nanjing, China). The 96-well polystyrene microtiter plate (MaxiSorp) was bought from Thermo Fisher Scientific (Shanghai, China). ASFV-positive freeze-dried serum, porcine negative serum national reference was purchased from the National Center of Veterinary Culture collection (CVCC) (Beijing, China).

### In vitro SELEX procedures

**Figure 8:**
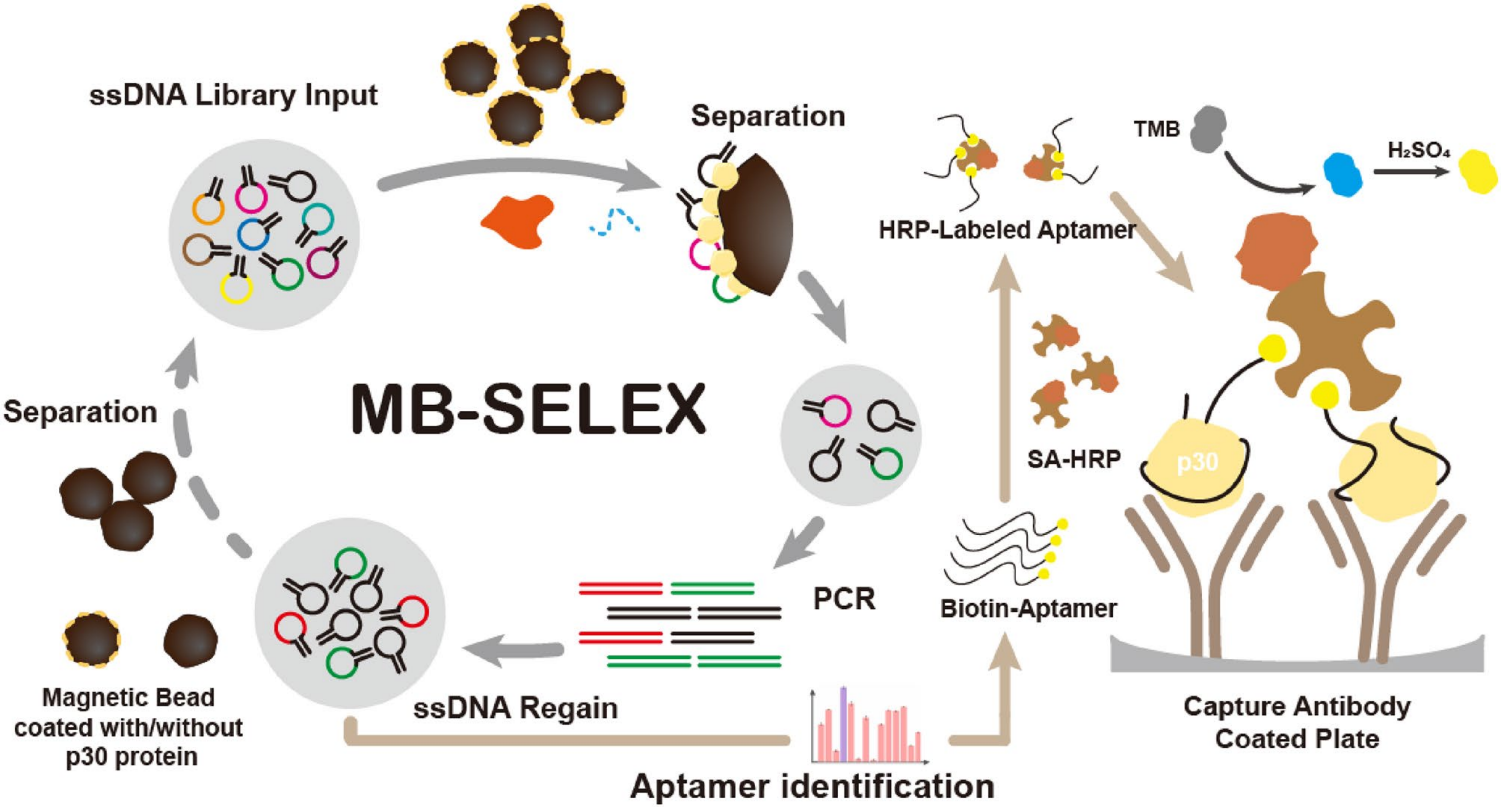
The flow chart of MB-SELEX strategy for specific aptamers against ASFV p30 protein and the principle of antibody/aptamer sandwich assay.

Target ASFV p30 proteins were immobilized on MB and incubated with a random oligonucleotide library for aptamer screening (Fig. 8).

NHS-MB contains *N*-hydroxysuccinimide functional groups, which can react with the primary amines on p30 proteins to form stable amide linkages. In this study, 190 μl p30 proteins (1 mg/ml) were reacted with 400 μl (equal to 4 mg) NHS-MBs to reach a relatively high protein immobilized capacity, showing an immobilization efficiency of 55% by BCA protein assay kit (Figure S2). The designed ssDNA library consisted of 40 nt oligonucleotide random sequences flanked with two primer sequences of 20 nt each. The sequence information is listed in Table S1. At the beginning of SELEX, 100 μl D-PBS buffer (0.01 M, pH 7.4) was added to the initial ssDNA library, which was heated at 95 °C for 10 min and cooled at − 20 °C for 5 min. The mixture was allowed to cool to room temperature to allow protein folding into three-dimensional structures. 1350 pmol ssDNA library was incubated with 50 μl of MBs (approximately to 40 μg p30 protein) at room temperature for 30 min at the initial round of SELEX; each input amount was gradually decreased at the next SELEX round. Additionally, NHS-MB containing no p30 protein and 6× His tag and swine serum were gradually added to the incubation solution as interference factors. The details are shown in Table S2. The unbound ssDNAs were magnetically separated in the supernatant after incubation, and MBs bound to aptamer candidates were washed with 0.01 M D-PBS and then eluted with ddH_2_O at 95 °C for 10 min. The elution solutions were collected for qPCR SELEX and the amplification qPCR program was conducted under the following conditions: 95 °C for 3 min; 20–35 cycles of 95 °C for 10 s, 60 °C for 30 s, 70 °C for 30 s. For aptamer candidate amplification and ssDNA regaining, elution solutions were added as templates to a PCR amplification system, which contained FAM-modified forward primer and poly-A modified reverse primer (all the sequence details are listed in Table S3). The PCR program was conducted under the following conditions: 95 °C for 3 min, 18–23 cycles of 95 °C for 30 s, 60 °C for 30 s, 72 °C for 60 s, and 72 °C for 4 min. Next, the PCR products were concentrated and loaded to 8% urea denaturing PAGE for ssDNA regain. After qPCR quantification, the obtained subordinate ssDNA libraries were input to the next round of SELEX until ssDNA library enrichment.

### Monitoring of aptamer screening

To determine the ssDNA concentration in SELEX, qPCR experiments were conducted on the initial ssDNA library at different concentrations (10^–3^–10^–8^ μM), and the quantitative curve was fitted based on the corresponding threshold cycle (cq). qPCR experiments were conducted, and the ssDNA input and elution amounts of rounds 1–7 (A_input_ and A_elution_) were calculated according to the quantitative curve. The retention ratio (R_e_) of each round was further calculated according to the following equation: ${R}_{e}={A}_{elution}/{A}_{input}$ for SELEX processes monitoring. In addition, amplification curves and melting curves from qPCR experiments were recorded for aptamer screening monitoring.

### Aptamer identification and binding affinity determination

The enriched ssDNA libraries were sent to Sangon Biotech Co., Ltd. (Shanghai, China) for library preparation and sequencing, and sequences with abundant repetitions were synthesized for further study. The binding affinity experiment was conducted similarly to the abovementioned target-protein binding step, where aptamer candidate sequences (each 100 pmol) were incubated with 40 μl coupled MBs. The corresponding elution ratios were calculated to compare the binding affinity. The secondary structures of these candidate sequences were analyzed using the web-based program M-fold (http://www.unafold.org/).

### Truncation of selected aptamer and measurement of the Dissociation Constant K_d_

Aptamers with the highest affinity were symmetrically truncated from the 3′ and 5′ ends according to their secondary structure. An enzyme-linked aptamer adsorbed assay was employed to determine the affinity. Firstly, the 96-well polystyrene microtiter plate was coated with 100 μl of p30 protein (5 μg/ml) at 4 °C overnight, and washed four times with 300 μl of PBST buffer (0.01 M, containing 0.05% Tween-20), followed by blocking with 100 μl of 1% BSA solution (10 g/l in 0.01 M PBS) at room temperature for 2 h. Secondly, the coated plate was loaded with 100 μl of truncated aptamer, which was modified with biotin and incubated at room temperature for 30 min. The unbound aptamer was washed three times with 250 μl of PBST buffer. Thirdly, 100 μl of SA-HRP (1:5000 diluted) were added to the plates for SA-Biotin reaction at RT for 30 min and then washed three times with 250 μl of PBST buffer to remove free SA-HRP. Finally, 100 μl of TMB substrate solution was added and reacted at 37 °C for 15 min, and the chromogenic reaction was terminated by adding 50 μl of stop solution. Absorbance was measured at 450 nm. For K_d_ measurement, the coated plate was loaded with varying concentrations (0–200 nM) of original aptamer and truncated aptamer to conduct the abovementioned enzyme-linked aptamer adsorbed assay. K_d_ was calculated according to the following nonlinear fitting formula: $A{\prime}={N}_{max}\times c/{K}_{d}+c$, where $A{\prime}$ was the absorbance of different concentrations of aptamer and c was the aptamer concentration.

### Antibody/enzyme-linked aptamer adsorbed assay for ASFV p30 protein detection

In order to simplify the detection procedures and improve analysis performance, the HRP-labeled aptamer was constructed and an ASFV p30 monoclonal antibody was employed as a capture element for p30. The concentration of biotin-aptamer and the diluted ratio of SA-HRP were optimized (details shown in S3). The optimal protocol was to mix the biotin-labeled Atc-20 with SA-HRP (1:1000 diluted) to achieve a final concentration of Atc-20 of 50 nM, which was then incubated for 90 min at 200 rpm in a shaker. For ASFV p30 protein capture, a 96-well polystyrene microtiter plate was coated with 100 μl ASFV p30 protein monoclonal antibodies (2 μg/ml) at 4 °C overnight. The ASFV p30 protein detection assay was then conducted as described above with slight modifications (Fig. 8). The p30 protein samples (50 μl) were loaded onto the coated plate, incubated at 37 °C for 2 h, and washed four times with 300 μl of PBST buffer. Next, 100 μl of prepared HRP-labeled aptamer was added and incubated for 40 min at 37 °C. Subsequently, the plates were washed three times with 300 μl of PBST buffer, and the chromogenic reaction was conducted unchanged. Absorbance was measured at 450 nm, and analytical standard curves were constructed by analyzing p30 protein of varying concentrations.

### Specificity and practicability analysis of the antibody/aptamer sandwich assay

In the specificity analysis, ASFV p30 protein of a concentration of 0.2 μg/ml and 2 μg/ml of other proteins were used. The mixture group consisted of 0.2 μg/ml and 2 μg/ml of other proteins. To demonstrate the practicability of this sandwich assay, the ASFV positive and negative serums were diluted 1, 2, 5, 10, and 100-fold with 0.01 M PBS. Three tenfold diluted serum samples were spiked with ASFV p30 standard solutions to achieve concentrations of 10, 20, and 40 ng/ml, respectively. The sample volume and experiment process were kept the same as above. The recovery ratios for the spiked ASFV p30 samples were calculated according to the formula $R=Cr/Ci$, where R was the recovery ratio of the spiked ASFV p30 samples, Cr was the detected ASFV p30 concentration of sandwich assay, and Ci was the spiked concentration of ASFV p30 samples. The concentrations were calculated according to the calibration curve of ASFV p30 protein.

### Supplementary Information

Supplementary Information.

## Data Availability

The sequencing datasets are available in the Sequence Read Archive (*SRA)* repository, [SRA data: PRJNA990511].
