# Supplementary material for: In vitro SELEX and application of an African swine fever virus (ASFV) p30 protein specific aptamer
Source: Sci Rep. 2024 Feb 19;14:4078. doi: 10.1038/s41598-024-53619-7 (PMC10876938; doi:10.1038/s41598-024-53619-7)
Supplement: Supplementary file 1 — Supplementary Information. [file 41598_2024_53619_MOESM1_ESM.docx]

**Supplemental materials**

**1. Table S1-S3**

**Table S1.** The proportion of top 14 sequences, summarized from sequencing results.

| **Name** | **Sequence 5’ to 3’** | **percentage** |
| --- | --- | --- |
| Apt 1 | ATTGGCACTCCACGCATAGGGGGTCGCTGGGAGTCTTGGAGGGAGGGTTACCCTGGATACCCTATGCGTGCTACCGTGAA | 9.27% |
| Apt 2 | ATTGGCACTCCACGCATAGGGTCGCCGGTCAGGCCGCCTTTGGTCCCAATCGTTGGGTGGCCTATGCGTGCTACCGTGAA | 7.40% |
| Apt 3 | ATTGGCACTCCACGCATAGGCCCGTTAGAAACTTGGAGGGAGAATCTGACGGCAGTTTGACCTATGCGTGCTACCGTGAA | 7.10% |
| Apt 4 | ATTGGCACTCCACGCATAGGGTCGACGGTGCCGTCCGTGTTCACACTCCCATTTGGGTGGCCTATGCGTGCTACCGTGAA | 6.75% |
| Apt 5 | ATTGGCACTCCACGCATAGGCTCTGGTCGGGAGTCTTGGAGGGAGGGTTCCCAACCGTCACCTATGCGTGCTACCGTGAA | 6.13% |
| Apt 6 | ATTGGCACTCCACGCATAGGGCACCAACCATGATGGGAGTCTTGGAGGGAGGGTTCCCATCCTATGCGTGCTACCGTGAA | 4.21% |
| Apt 7 | ATTGGCACTCCACGCATAGGACCGCAGCTCGCCGCCATTTATTCCCTGTCATAGGGTGTCCCTATGCGTGCTACCGTGAA | 4.12% |
| Apt 8 | ATTGGCACTCCACGCATAGGGGGTCCGGGGTGGTGTAGGGGGTTAGGGTGGGCTTTACGACCTATGCGTGCTACCGTGAA | 3.74% |
| Apt 9 | ATTGGCACTCCACGCATAGGGGCCATGGGAGTCTTGGAGGGAGGGACCCGGTTTGCGGGACCTATGCGTGCTACCGTGAA | 3.30% |
| Apt 10 | ATTGGCACTCCACGCATAGGACTGCATAGGATACTTGGAGGGAGGTGTTCCTGGTTATGGCCTATGCGTGCTACCGTGAA | 2.88% |
| Apt 11 | ATTGGCACTCCACGCATAGGCGTCTCAGATGGGAGATACTTGGAGGGAGGGGCCCCATGGCCTATGCGTGCTACCGTGAA | 2.28% |
| Apt 12 | ATTGGCACTCCACGCATAGGCCGGCTTTAACGTGGATGCTTGGAGGGAGGTTCCGCTGCCCCTATGCGTGCTACCGTGAA | 2.06% |
| Apt13 | ATTGGCACTCCACGCATAGGGGTGCTTTGGGAAACTTGGAGGGAGGGCCTCCCGGGGCCCCCTATGCGTGCTACCGTGAA | 1.92% |
| Apt 14 | ATTGGCACTCCACGCATAGGTCGGGCATACGGGATACTTGGAGGGAGGGTTCCCGCACAGCCTATGCGTGCTACCGTGAA | 1.55% |

The datasets generated during the current study are available in the Sequence Read Archive (SRA) repository, [SRA data: PRJNA990511]

**Table S2.** The parameters of SELEX. NHS-MB contained no p30 protein as NC-MB was input starting from the 4th round, the nonspecific aptamers bound with NC-MB were removed by magnetic separator.

| **SELEX Round** | **Pool/pmol** | **Bead/*μ*L** | **Incubation time/min** | **His-tag/*μ*g** | **Serum/*μ*L** | **Retention rate** |
| --- | --- | --- | --- | --- | --- | --- |
| 1 | 1350 | 50 | 30 | 0 | 0 | 0.0031% |
| 2 | 135 | 50 | 30 | 0 | 0 | 0.0219% |
| 3 | 135 | 40 | 30 | 0.5 | 0 | 2.6506% |
| 4 | 67.5 | 40 | 20 | 1 | 1.5 | 0.0170% |
| 5 | 67.5 | 40 | 20 | 1.5 | 1.5 | 2.9246% |
| 6 | 67.5 | 40 | 20 | 2.5 | 3 | 5.8280% |
| 7 | 67.5 | 40 | 20 | 3.5 | 5 | 5.3181% |

**Table S3.** The designed ssDNA library consisted of 40 nt oligonucleotides random sequences and was flanked with two primers sequences of each 20 nt. Primers A and B were used in qPCR experiments; primers C and D were used in PCR amplification experiments.

| **Name** | **Sequence 5’-3’** | **Modification** |
| --- | --- | --- |
| Initial library | ATTGGCACTCCACGCATAGG(N)_40_CCTATGCGTGCTACCGTGAA | \ |
| Primer A | ATTGGCACTCCACGCATAGG | \ |
| Primer B | TTCACGGTAGCACGCATAGG | \ |
| Primer C | ATTGGCACTCCACGCATAGG | 5’- FAM |
| Primer D | AAAAAAAAAAAAAAAAAAAAAAAAA/iSp18/TTCACGGTAGCACGCATAGG | Spacer-18 |
| Apt A4 | ATTGGCACTCCACGCATAGGGTCGACGGTGCCGTCCGTGTTCACACTCCCATTTGGGTGGCCTATGCGTGCTACCGTGAA | 5’-Biotin |
| Atc-20 | CACGCATAGGGTCGACGGTGCCGTCCGTGTTCACACTCCCATTTGGGTGGCCTATGCGTG | 5’-Biotin |
| Atc-28 | CATAGGGTCGACGGTGCCGTCCGTGTTCACACTCCCATTTGGGTGGCCTATG | 5’-Biotin |
| Atc-34 | GGGTCGACGGTGCCGTCCGTGTTCACACTCCCATTTGGGTGGCC | 5’-Biotin |
| AptNC | ATTGGCACTCCACGCATCTATGCGTGCTACCGTGAA | 5’-Biotin |

1. **Figure S1-S3**
2.
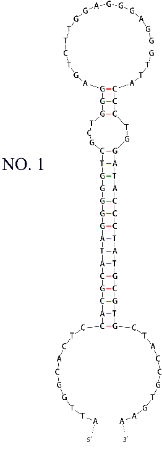

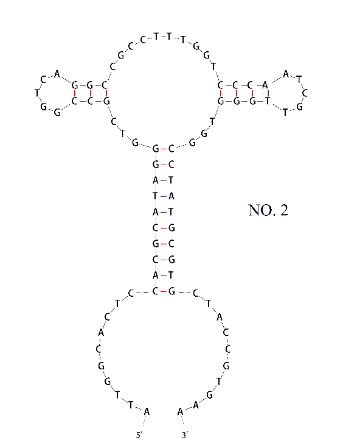

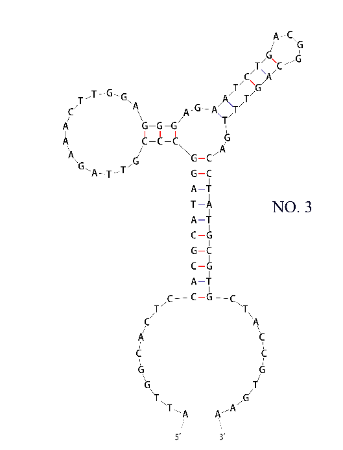

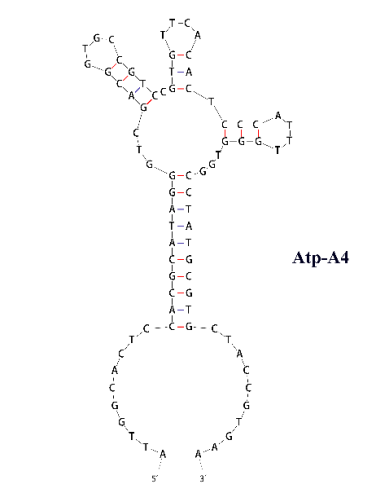

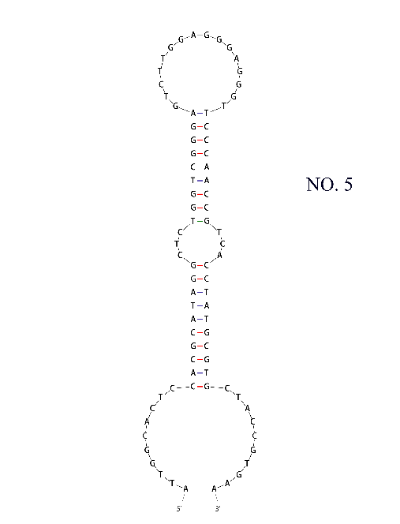

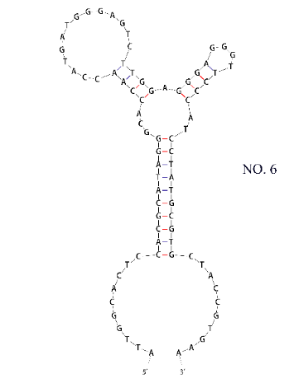

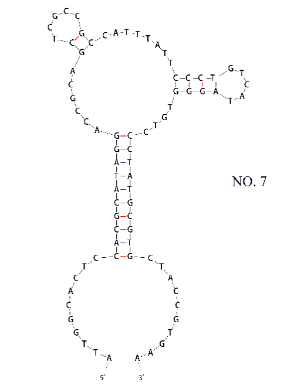

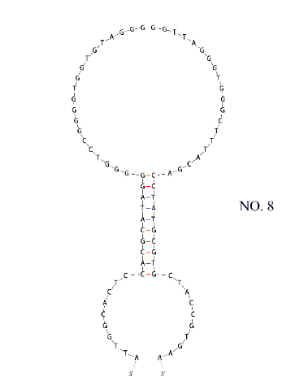

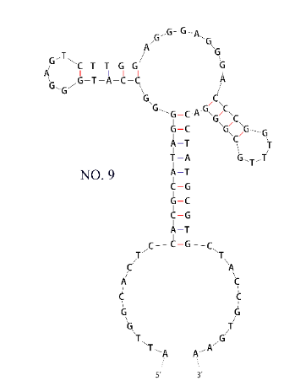

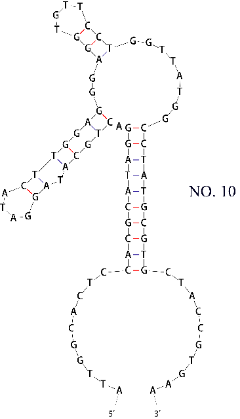

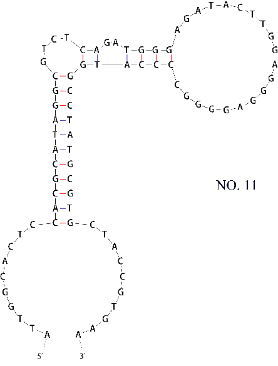

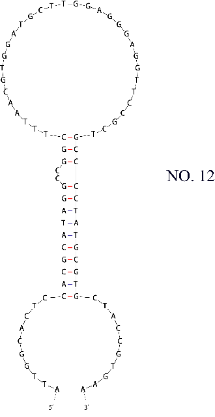

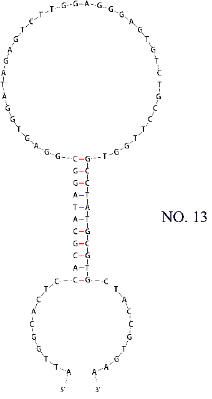

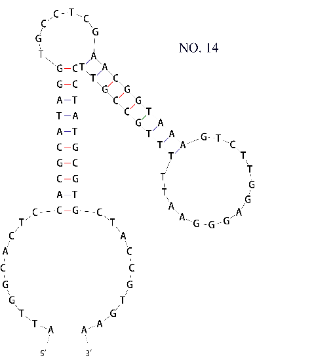


**Figure S1.** The predicted second structures of Apt A1-14.

**1.1** In BCA protein assay kit, the standard BSA solution was used to construct Standard curve for protein concentration quantification. The linearly relation of protein concentration and A_562_ (Absorbance in 562 nM) was shown in Figure S2. Employing BCA assay, the immobilization efficiency was calculated by $E=1-\frac{C1}{C0}$, where E is the immobilization efficiency; C1 is the protein concentration of magnetic separated supernatant; C0 is the protein concentration of initial protein solution.

**

**

**Figure S2.** Standard curve of BCA protein assay kit.

**1.2** Proportional optimization of the HRP-labeled aptamer preparation

To determine the approximate range of the dilution and the concentration, the SA-HRP were first diluted 250, 500, 1000, 2000, 40000-fold with 0.1 M PBS and fully shaking, afterward, Biotin-labeled Atc-20 (10 *μ*M) were added to achieve the final concentrations of 50, 100, 150, 200, and 500 nM and incubated in a shaker for 2 h (200 rpm, RT). The 96-well plates were coated with 100 *μ*L of p30 monoclonal antibody (2 *μ*g/mL) over night at -4 ℃. After blocking and washing, 50 *μ*L of ASFV p30 protein (0.2 *μ*g) was added and incubated at 37 ℃ for 2 h, whereafter, 100 *μ*L of HRP-labeled Atc-20 of different proportions were added and incubated for 40 min to bind with p30 protein. 100 *μ*L TMB substrate solutions were added for the chromogenic reaction. Finally, 50 *μ*L of stop solutions were added, and the absorbance was measured at 450 nm. As shown in Figure S1(a), the absorbance increased overall, following the decreased SA-HRP dilution ratio. However, the increased concentration of biotin-aptamer failed to enhance signal intensity effectively, or even exhibited lower signal intensity at 200 and500 nM concentrations than 50 and 100 nM concentrations. On the basis of pre-experiment, we further optimized the dilution ratio of SA-HRP at 50 and 100 nM Biotin-labeled Atc-20 concentrations. The HRP-labeled aptamer preparation and other experimental procedures were carried out as described above. The resultes are shown in Figure S3(b), and the Biotin-labeled Atc-20 at 50 nM exhibited higher signal intensity than 100 nM at different SA-HRP dilution ratios. However, the blank signal intensity gradually increased following the decreased SA-HRP dilution ratio. We calculated the signal to noise ratio (S/N) as shown in figure S3(a) insert, the dilutuin ratio at 1:1000 exhibited highest signal to noise ratio, and thus the dilution was selected at 1:1000 as the optimal dilution ratio. The prepared HRP-labeled aptamer can be stored at 4 ℃ before use, which reduces testing time by at least 40 minutes.







**Figure S3**. (a) Different concentrations of biotin-aptamer and dilute rates of SA-HRP (b) Biotin-Aptamer at concentrations of 50, 100 nM were incubated with SA-HRP at the dilution rates ranging from 1:250 to 1:4000
